# Compositional Learning of Human Activities With a Self-Organizing Neural Architecture

**DOI:** 10.3389/frobt.2019.00072

**Published:** 2019-08-27

**Authors:** Luiza Mici, German I. Parisi, Stefan Wermter

**Affiliations:** Department of Informatics, Knowledge Technology, University of Hamburg, Hamburg, Germany

**Keywords:** human activity recognition, self-organizing networks, hierarchical learning, compositionality of human activities, RGB-D perception

## Abstract

An important step for assistive systems and robot companions operating in human environments is to learn the compositionality of human activities, i.e., recognize both activities and their comprising actions. Most existing approaches address action and activity recognition as separate tasks, i.e., actions need to be inferred before the activity labels, and are thus highly sensitive to the correct temporal segmentation of the activity sequences. In this paper, we present a novel learning approach that jointly learns human activities on two levels of semantic and temporal complexity: (1) transitive actions such as *reaching* and *opening*, e.g., a cereal box, and (2) high-level activities such as *having breakfast*. Our model consists of a hierarchy of GWR networks which process and learn inherent spatiotemporal dependencies of multiple visual cues extracted from the human body skeletal representation and the interaction with objects. The neural architecture learns and semantically segments input RGB-D sequences of high-level activities into their composing actions, without supervision. We investigate the performance of our architecture with a set of experiments on a publicly available benchmark dataset. The experimental results show that our approach outperforms the state of the art with respect to the classification of the high-level activities. Additionally, we introduce a novel top-down modulation mechanism to the architecture which uses the actions and activity labels as constraints during the learning phase. In our experiments, we show how this mechanism can be used to control the network's neural growth without decreasing the overall performance.

## 1. Introduction

The successful application of robots as companions and assistive systems requires a reliable perception of the surrounding environment. Beyond the analysis of the structure of the environment and the recognition of the present objects, the full understanding of human behavior is a key component for such applications (Koppula and Saxena, 2013; Vrigkas et al., 2015; Tianmin Shu and Zhu, 2016). An interactive robot system should be aware of the assisted person's daily activities in order to assess his/her well-being and to plan future actions accordingly.

**Figure 1:**
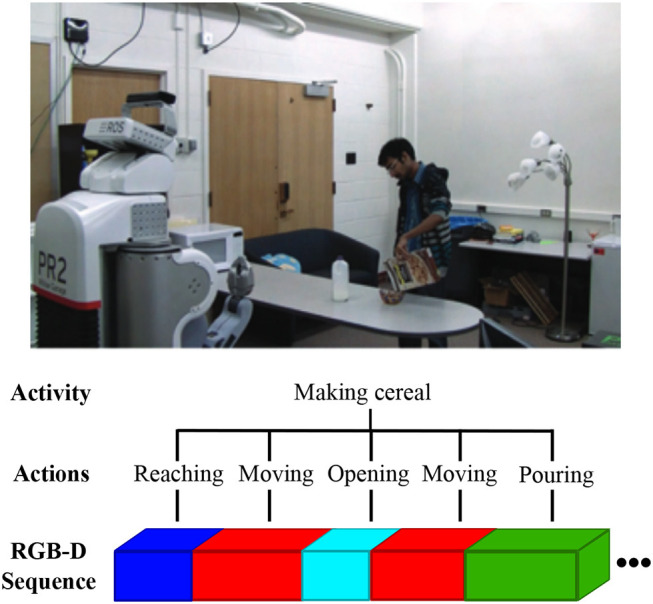
An illustration of the hierarchical compositionality of human activity visually perceived by a robot (the RGB image belongs to the CAD-120 dataset Koppula et al., 2013). The input to the robot is an RGB-D sequence including the spatio-temporal dependencies of the human-object interaction. The sequence represents a high-level human activity which can be semantically segmented into smaller composing actions of varying lengths.

Human daily actions and activities are diverse and complex. The same type of action can be performed in many different ways and with different objects, e.g., a person can drink from a cup or a bottle, he/she can sit on a chair but also on the floor. Even a small set of actions and objects can create a large combination of possible activities. Additionally, human activities can achieve similar goals through a varying combination of smaller actions, e.g., the activity of *making cereal* can include actions such as *pouring milk, pouring cereal*, and *opening* or another set of actions according to the person's daily routine (see Figure 1). The representation of actions and goals in the human brain are ordered hierarchically according to their level of abstractness or time of completion. We distinguish mainly between simple movements (e.g., opening the hand), actions (often transitive actions, e.g., reaching or grasping a cookie), immediate goals (e.g., take a cookie) and task goals (e.g., prepare a snack; Hamilton and Grafton, 2006). A task goal may involve several immediate goals, to be achieved through a sequence of actions. Finally, each action is composed of several movements. Learning approaches can benefit from a similar hierarchical modeling of human activities in order to anticipate task goals. Moreover, the semantic compositionality analysis of the human activities is beneficial for potential robotic applications such as the detection of forgotten steps in a perceived sequence of human actions, e.g., detecting that a person has forgotten to put the milk back in the fridge after having meal (Wu et al., 2015). On a more abstract level, through the compositional learning of daily human-object interactions, autonomous robots can gather knowledge about object *affordances* and possible task execution strategies which can be used to generate potential plans given a goal to be reached (Kjellström et al., 2011; Pieropan et al., 2014a; Jamone et al., 2016).

Despite the considerable progress on the recognition of the human actions in the recent decade, the question remains open on how to efficiently model and represent the rich hierarchical structure of human-object interactions (Aggarwal and Xia, 2014). Quite often, the perceptual sequences learned at the lower level are combined into more complex sequences, or activities, by assigning them arbitrary symbols or rules (Wermter, 2000; Taniguchi et al., 2016). Such symbols are usually fixed and defined a priori by the designers based on their domain knowledge making the system less adaptable to previously unseen sequences or action-object combinations. Moreover, most of the previous work on the hierarchical recognition of human activities addresses activity and action recognition as separate tasks (Koppula and Saxena, 2013; Koppula et al., 2013), i.e., the action labels need to be inferred before the activity labels. The activity classification accuracy of such approaches is sensitive to the correct classification of the composing actions.

In contrast to these approaches, in this work we seek to jointly model actions and activities with one hierarchical learning framework, whereby spatiotemporal dependencies of human-object interactions are learned and stored as neural representations. More formally, we propose a hierarchical arrangement of Growing When Required (GWR) networks (Marsland et al., 2002) which integrate multiple visual cues regarding the body pose, the manipulated object, and their spatial relation during human-object interaction, accumulated over a short and a longer period of time in order to jointly learn actions and human activities respectively. In our previous research, we have successfully applied and evaluated hierarchical architectures of the GWR network for clustering human body pose and motion patterns as well as for learning prototypical representations of human-object interactions in an unsupervised fashion (Parisi et al., 2015; Mici et al., 2018a,c). The generative properties of the GWR networks have been shown to be particularly suitable for the human-object interaction recognition due to generalizing well to unseen action-object pairs.

Similar to our previous work, we will make use of the three-dimensional human skeleton and RGB-D features that can be obtained through low-cost and non-invasive RGB-D sensors, such as the Microsoft Kinect and Asus Xtion Pro cameras. Moreover, skeleton body representations have been successfully applied to the recognition and prediction of human-object interactions (Mici et al., 2018b) as well as to the problem of compositional learning of human-object interactions (Wu et al., 2015). We evaluate our model by running a set of experiments with the publicly available benchmark dataset CAD-120 (Koppula et al., 2013), which allows for a compositionality analysis of human daily activities. Experimental results show that we outperform the state-of-the-art approaches with respect to the recognition of high-level activities. A qualitative analysis of the labels generated by the architecture during the test phase shows that semantically meaningful representations of the composing actions emerge.

The current work is novel in two main aspects: First, the proposed architecture can jointly learn in an unsupervised manner two levels of semantic and temporal complexity of human actions, namely actions, and high-level activities that can be composed of different actions. Second, we propose a top-down modulation mechanism which uses the action and activity labels to modulate the neural insertion of the hierarchical architecture during the learning phase. Through such a mechanism, the neural representations of actions and activities are optimized according to the classification error rather than according to the error of the input representation.

The rest of this paper is structured as follows. In section 2, we describe related work addressing the compositional learning of human-object interactions from video data. In section 3, we present our hierarchical self-organizing architecture and the learning GWR algorithm extended with a top-down modulation mechanism. In section 4, we provide experimental results on the CAD-120 dataset. We conclude in section 5 and point toward possible future work directions.

## 2. Related Work

The recognition of human activities requires learning complex spatiotemporal relationships between features of human body actions and manipulated objects. Depending on the complexity and duration of the activities, recognition approaches can be separated into two categories: single-layer approaches and hierarchical approaches (Aggarwal and Ryoo, 2011). Single-layer approaches refer to methods that infer human activities directly from the data without defining any activity hierarchy. Typical hierarchical approaches, on the other hand, first estimate simple actions, and then, the high-level activity labels are inferred based on the action sequences.

### 2.1. Single-Layer Approaches

A great number of single-layer approaches address simple and short actions, such as walking, jumping, and falling. The study of hand-actions, such as grasping, placing and holding, has also received particular interest in robotics in order to accomplish the recognition of robotic grip apertures and the learning of affordances (Prevete et al., 2008; Tessitore et al., 2010; de Jesús Rubio et al., 2018).

Various approaches for the recognition of human-object interactions do not explicitly model the interplay between object recognition and body pose estimation. Typically, objects are first recognized and activities involving them are subsequently recognized by analyzing the objects' motion trajectories (Wu et al., 2007) or by considering possible language trigrams <*Object1, Action, Object2*> extracted from English sentences (Yang et al., 2015). Pieropan et al. (2014b) proposed including action-related audio cues in addition to the spatial relationship among objects in order to learn object manipulations for the purpose of robot learning by imitation. However, important descriptive visual features like body motion or fine-grained cues like the hand pose during manipulation were not considered.

Probabilistic approaches have been extensively used for reasoning upon relationships and dependencies among objects, motion, and human activities such as hidden Markov Models (HMM) and Bayesian networks (Gupta et al., 2009; Kjellström et al., 2011). Other research studies have modeled the mutual context between objects and human pose through graphical models such as the Conditional Random Fields (CRF) (Kjellström et al., 2011; Yao and Fei-Fei, 2012). These types of models suffer from high computational complexity and require a fine-grained segmentation of the action sequences. Other approaches extract novel low-level visual features encoding the spatial relationships between the human and the manipulated objects, such as the *Grouplet* feature proposed by Yao and Fei-Fei (2010). Their method is able to distinguish between interactions or just co-occurrences of humans and objects in an image, but no applications to video data have been ireported.

Neural network models have also been successfully applied for the problem of understanding human-object interactions from visual sensory input. Shimozaki and Kuniyoshi (2003) proposed a hierarchical architecture based on the self-organizing maps (SOMs) capable of integrating object categories, spatial relationships, and movement. The architecture was shown to perform well on simple 2D scenes of ball-handling actions. However, compared to the static image domain, there is limited work on understanding human-object relationships from video data sequences with neural network architectures (Lea et al., 2016; Ma et al., 2017).

Since the introduction of the low-cost depth sensing devices such as Microsoft Kinect and Asus Xtion, there has been extensive work in human action recognition from depth data (Sung et al., 2012; Yang and Tian, 2014; Cippitelli et al., 2016). From a large number of human body representation approaches we can distinguish between two broad categories: (1) representations based on the RGB-D information, and (2) representations based on the 3D skeleton data. Methods belonging to the first category compute, for instance, the temporal evolution of the body 3D silhouettes during action performance (Li et al., 2010; Yang et al., 2012), or use the STIP descriptors which are invariant to spatiotemporal shifts and scales and can deal with body occlusions during human-object interactions. However, 3D silhouette-based algorithms are usually view-dependent while the STIP-based methods require the whole video as input and are very slow to compute, thus limiting their real-time application. High computational cost and poor real-time performance is also the major limitation of approaches based on 3D optical flow or scene flow using RGB and depth (see Aggarwal and Xia, 2014 for a review).

Unlike the features from 3D silhouettes, the skeletal joint features are invariant to the camera location and subject appearance or to body size. Human action recognition methods based on skeletal joints have been successfully applied in real time in order to recognize finer human-object interaction activities than 3D silhouette-based approaches (Aggarwal and Xia, 2014). The main limitation of the skeletal body representation is the lack of information about surrounding objects. For this, Wang et al. (2014) proposed a new 3D feature, named local occupancy pattern (LOP), which computes the local occupancy information based on the 3D point clouds surrounding a 3D joint. In this way, an LOP feature can capture the relations between the human body parts, e.g., hands, and the objects that the person is interacting with. Although this method produced state-of-the-art results, the identity of the manipulated objects is completely ignored, and it is unclear how much discriminative are the 3D features when objects are small and partially occluded. Alternatively, other methods model human-object interactions considering the skeletal features combined with the object's identity (Rybok et al., 2014; Mici et al., 2018c).

### 2.2. Hierarchical Approaches

One of the earliest approaches toward hierarchical recognition of human activities was proposed by Ryoo and Aggarwal (2007). The author modeled motion patterns with HMMs and introduced an additional semantic layer providing feedback to the modules for object identification and motion estimation leading to an improvement of object recognition rates and better motion estimation. Nevertheless, the subjects' articulated body pose was not considered as input data, leading to applications in a restricted task-specific domain such as airport video surveillance. Aksoy et al. (2017) proposed the *Semantic Event Chain* (SEC) concept, i.e., a matrix whose entries represent the spatial relationship between extracted image segments for every video frame. Action classification is obtained in an unsupervised way through maximal similarity. While this method is suitable for teaching object manipulation commands to robots, the representation of the visual stimuli does not allow for reasoning upon semantic aspects such as the congruence of the action being performed on a certain object.

A number of approaches for learning the hierarchical representations of human activities first segment and classify actions and then infer the high-level activities based on the action sequences. Hybrid approaches, for instance, learn perceptual sequences, e.g., through a neural network model, at the lower level and combine them into more complex sequences, or activities, by assigning them arbitrary symbols or rules (Wermter, 2000; Taniguchi et al., 2016). Their main limitation is the fixed set of symbols which is usually pre-defined based on the designers' domain knowledge. More related to our approach is the work from Wu et al. (2015), in which the *k-means* algorithm is used to cluster video data of human activities and discover the so-called *action-words*. Then, the video sequences are represented as sequences of action-words and an unsupervised model is trained on them to learn action co-occurrence and action temporal relations. The authors, however, do not provide experimental results on the compositional learning of the human activities, but rather focus on the actions co-occurrence and on detecting a forgotten action in a perceived action sequence. Koppula and Saxena (2013) presented a CRF for learning the hierarchical compositionality of human activities by modeling the temporal and spatial relations between humans and manipulated objects. In contrast to their approach, in this paper, we propose a hierarchical model that jointly learns actions and activities. In our neural architecture, the correct classification of high-level activities is not sensitive to the correct segmentation and classification of the composing actions.

## 3. Methodology

**Figure 2:**
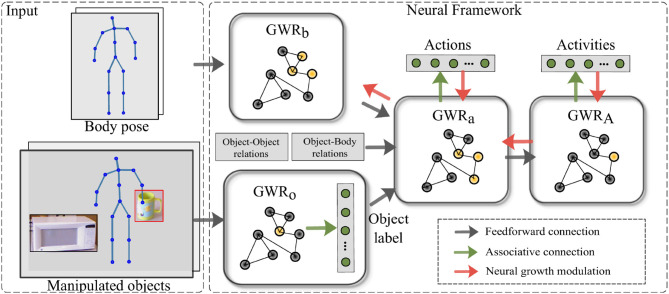
Overview of the proposed architecture (Mici, 2018). The *GWR*_*b*_ and *GWR*_*o*_ networks process low-level features extracted from the skeletal body representation and the RGB images of the manipulated objects, respectively. Additional visual features are extracted to capture the body-object and object-object spatial relationships. The *GWR*_*a*_ and *GWR*_*A*_ networks learn spatiotemporal dependencies of the human-object interactions over two different time windows. Both networks have associative connections to two symbolic layers holding information about actions and activities. The top-down modulation mechanism, when enabled, modulates the learning of the *GWR*_*b*_, *GWR*_*a*_, and *GWR*_*A*_ networks.

We propose a self-organizing hierarchical architecture for learning human actions on two levels of semantic and temporal complexity: (1) *actions* such as *reaching* or *opening* which are completed in a relative short period of time, and (2) the high-level *activities* that can be composed of different actions. An overall diagram of the architecture is shown in Figure 2.

The architecture consists of two main network streams processing separately visual representations of the body postures and of the objects being manipulated. The advantage of having two distinct network streams for learning prototype body pose and prototype objects is 2-fold: (1) it leads to greater generalization capabilities of the architecture in terms of recognizing unseen action-object pairs (Mici et al., 2018c), and (2) it attenuates to some extent the noise in the tracked body skeleton sequences (Parisi et al., 2015). For the perception of the body pose and motion, we rely on three-dimensional articulated body tracks provided in real time by depth sensor technologies, such as the Asus Xtion camera. For classifying objects, on the other hand, we consider the RGB object images which supply richer representations such as texture.

The *GWR*_*b*_, *GWR*_*o*_, and *GWR*_*a*_ networks process and subsequently integrate the body pose and the information about the manipulated object(s), while the *GWR*_*A*_ network integrates spatiotemporal dependencies over longer time windows and learns to classify human activities. Both the *GWR*_*a*_ and the *GWR*_*A*_ networks capture different temporal ranges of actions by the accumulation of body movement patterns over a short and a longer time period respectively. The feedforward hierarchical computation of the spatiotemporal inputs will be introduced in section 3.3. Besides the identity of the manipulated objects, we consider additional visual features capturing the object-object and object-body spatial relations, as will be described in section 4.2.

Additionally, we introduce delayed feedback connections and extend the traditional GWR learning algorithm with a top-down modulation mechanism. Thus, during training, the error regarding the misclassification of the actions and of the activities is propagated not only to the *GWR*_*a*_ and *GWR*_*A*_ respectively but also to the network layers preceding them (the feedback connections are depicted with red arrows in Figure 2). This is done in order to (1) allow changes of the topological structures for all the body processing GWR networks, and (2) better match the input space in order to jointly learn the actions and the high-level activities. We apply the proposed neuron insertion strategy to each network layer. Nevertheless, at the current state, the action classification error is not propagated to the object recognition module which provides the identity of the manipulated objects at the beginning of each action sequence.

### 3.1. Growing When Required Networks

The building block of our neural framework is the Growing When Required network (GWR) proposed by Marsland et al. (2002). The GWR algorithm is a growing extension of the SOMs which learns to represent the input data distribution through a finite set of prototype neurons. During learning, the prototype neurons adaptively form topology preserving maps of the input space in an unsupervised fashion, i.e., similar inputs are mapped to neurons that are near to each other on the map.

The GWR network has two main components: the nodes associated with a weight vector and the edges that link the nodes to form neighborhood relationships. The dynamics of the network are defined by two main steps: (1) the competition among the neurons for representing an input data sample, and (2) the adaptation of the network's topology toward the input space. The first step uses a similarity measure, namely the Euclidean distance, between an input data sample **x**(*t*) and the weight vector of each neuron in the network. Thus, the index of the best-matching unit (BMU) at time step *t* is given by:

(1) $$\begin{array}{r} b=arg\underset{j\in A}{\min}||\text{x}\left(t\right)-{\text{w}}_{j}||, \end{array}$$

where **w**_*j*_ is the weight vector of the *j*th neuron and *A* is the set of all weight vectors. The topology adaptation step affects the BMU as well as all neurons that have established a neighborhood relationship with the BMU according to the structure of the network.

In the initial state, the network consists of a set of two nodes randomly initialized from within the training data. Both nodes and edges can be created and removed during each learning iteration. The network growth rate is a function of the overall network activation with respect to the input, which is computed as a function of the Euclidean distance between the weight of the best-matching unit, **w**_*b*_ and the input data sample **x**(*t*) at time step *t*:

(2) $$\begin{array}{r} a\left(t\right)=\exp\left(-||\text{x}\left(t\right)-{\text{w}}_{b}||\right). \end{array}$$

The output of the activation function is equal to 1 when the BMU perfectly matches the input, i.e., the Euclidean distance between the weight of the BMU and the input is 0, and it decays exponentially toward 0 for greater distances. When the activity of the best-matching unit is lower than a predefined threshold, named insertion threshold *a*_*T*_, new neurons will be inserted between the BMU and the input. The insertion threshold parameter modulates the amount of generalization, i.e., the discrepancy between an incoming stimulus and its best-matching unit.

Edges are created by applying the competitive Hebbian learning method (Martinetz, 1993), i.e., they are generated between the two nodes with the smallest distance from the input data sample. As a consequence, after several learning iterations, two nodes with an existing edge may result far from each other, thereby not representing similar perceptions. An aging mechanism together with a threshold *a*_*max*_ takes care of removing such edges and unconnected, hence redundant, nodes consequently. Additionally, a firing counter mechanism named *habituation*, *h*, measures how often each node has fired. Every time a neuron matches the input data sample, its habituation is updated following (Equation 8) in Algorithm 1. The habituation is part of the neuron weight update equation in a way that nodes that have fired frequently are trained less (see Equation 7 in Algorithm 1). This learning mechanism affects the network's local adaptations at each learning iteration and leads to a decreased response of the network to a stimulus that has been frequently presented.

### 3.2. Classification

While keeping the learning process unsupervised, the GWR algorithm can be extended with a labeling strategy in order to solve classification tasks as well (Parisi et al., 2017b; Mici et al., 2018b). Similar to our previous work, we apply a labeling method based on the majority vote strategy.

We link each neuron to a symbolic action label *l* ∈ *L*, where *L* is the set of action classes. The GWR neurons will then have a many-to-many relationship with the symbolic layer. The set of weights Π, which are initialized to zero, are updated according to a Hebbian learning rule:

(3) $$\begin{array}{r} \Delta \pi_{il_{j}}=\gamma \cdot a_{i}\left(t\right), \end{array}$$

where 0 < γ < 1 is the learning rate, *a*_*i*_(*t*) is the activity of the winner neuron at time step *t* and *l*_*j*_ is the target action label. After the training phase is complete, the weights are normalized by scaling them with the corresponding inverse class frequency and with the inverse neuron activation frequency. In this way, class labels that appear less during training are not penalized, and the vote of the neurons is weighted equally in spite of how often they have fired.

At recognition time, given one temporal segment of a human-object interaction at the time step *t*, the best-matching unit *b* is computed following (Equation 1) and the action label is given by:

(4) $$\begin{array}{r} l_{j}=arg\;\underset{l\in L}{\max}\left(\pi_{b,l}\right). \end{array}$$

In order to classify an entire action sequence, a majority vote labeling technique is applied on the labels of its composing temporal segments.

### 3.3. Feedforward Sequence Processing

In order to process and integrate the spatiotemporal information about the human body pose and the manipulated objects, we apply a hierarchical learning approach, whereby the output of each sequence processing GWR network is augmented with a *window-in-time* memory (Barreto, 2007; Parisi et al., 2017b).

In order to do so, we compute the neural activations of the *GWR*_*b*_, *GWR*_*a*_, and *GWR*_*A*_ networks and apply the delay embedding technique (Takens, 1981). We take the weight vectors of the BMUs over time and group them into vectors of the form:

(5) $$\begin{array}{r} \text{o}\left(t\right)=\left\{{\text{w}}_{b\left({\text{x}}_{i}\right)},{\text{w}}_{b\left({\text{x}}_{i-\xi }\right)},\ldots {\text{w}}_{b\left({\text{x}}_{i-\left(q-1\right)\xi }\right)}\right\},i\in \left[q,k\right], \end{array}$$

where *k* is the total number of training frames and *q* and ξ are the embedding parameters denoting the width of the time window and the lag or delay between two consecutive frames respectively. The choice of the embedding parameters are data-dependent and can be set following a heuristic method or, as in our case, can be chosen empirically. Moving up in the hierarchy, the output **o**(*t*) will represent the input for the GWR network of the higher layer. In this way, the *GWR*_*b*_ network learns a dictionary of prototypes of the spatial body configurations domain, while the *GWR*_*a*_ and *GWR*_*A*_ networks encode human-object interaction prototype segments accumulated over a short and a longer period of time respectively.

Objects are classified only at the beginning of an activity sequence. Therefore, the object representations to be learned contain no temporal information and the computation of the output reported in Equation (5) is not performed for the *GWR*_*o*_ network. The label of the *GWR*_*o*_ BMU is represented in the form of one-hot encoding, i.e., a vectorial representation in which all elements are zero except the ones with the index corresponding to the recognized object's category. When more than one object is segmented from the scene, the object data processing and classification with *GWR*_*o*_ is repeated as many times as the number of additional objects. The resulting labels are merged into one multiple-hot-encoded vector for the following integration step. This vector is then concatenated with the visual features representing object-object and object-body relationships and with the output of the *GWR*_*b*_ before being given as input to the *GWR*_*a*_ network.

### 3.4. A Top-Down Modulation Approach

As described in section 3.1, the insertion criterion of new neurons in the original GWR algorithm is decided based on the local representation errors of the network. If the activity of the BMU at time *t*, *a*(*t*), is lower than the insertion threshold *a*_*T*_, then a new neuron will be inserted. However, when target labels are available, the fact that the habituated BMU has been assigned a different label than the input it matches at time step *t*, for instance, can indicate that a new neuron should be inserted near the existing one. With this argument in mind, we take the local classification error information into consideration and introduce a new neuron insertion strategy which acts as a top-down training modulation mechanism.

The GWR algorithm decides the moment and place for the insertion of a new neuron at each learning iteration. For this reason, we equip each neuron with a way of measuring how often it has misclassified. We associate each neuron *i* with a counter *c*_*i*_, which is incremented whenever that neuron is the BMU of an input with a different label. Whenever the misclassification counter *c*_*b*_ of the habituated BMU at time step *t* exceeds a threshold *m*_*T*_, a new neuron will be inserted between the badly matched winning neuron and the input and will take the label of the input. If there is no mismatch between the input and the BMU, then the algorithm will proceed normally with the weight updates (see Algorithm 1).

As can be seen in Algorithm 1, step 6, we combine both quantization error with the classification error for the neuron insertion strategy. This would allow for the higher density of neurons in the regions where most misclassifications occur while guaranteeing that, at least, all the training data have a good prototype representation. Moreover, the use of the two conditions can serve as a stopping criterion for the learning process, i.e., when the network has learned to represent the input data in the best way possible, the growth will stop even though misclassifications may still take place. It should be noted that the sensibility of the network's growth with respect to the value of the insertion threshold parameter is more relaxed. Finding an optimal value for this parameter is no longer necessary for maximizing the classification performance of the model as long as both insertion conditions are used. Regarding the misclassification threshold parameter, setting the *m*_*T*_ to 0 is equivalent to having a GWR network where neural growth occurs as soon as misclassifications occur for a habituated neuron. A high threshold, on the other hand, causes the slow-down of the growth of the network and the available resources might not be enough to solve the classification task.

**Algorithm 1:** The GWR algorithm extended with the new neuron insertion strategy

Create a set *A* of two neurons at random positions. Initialize an empty set of connections *C* = ∅. (**Introduced symbolic connections**) Initialize the set of symbolic weights Π to zero. At each iteration *t*, generate an input sample **x**(*t*) with label **y**(*t*). Select the best and second-best matching unit as introduced in Equation 1: (6) $$\begin{array}{r} \begin{array}{c} b=arg\underset{n\in A}{\min}||\text{x}\left(t\right)-{\text{w}}_{n}||,\\ s=arg\underset{n\in A/\left\{b\right\}}{\min}||\text{x}\left(t\right)-{\text{w}}_{n}||. \end{array} \end{array}$$ Create a connection *E* = *E*∪{(*b, s*)} if it does not exist and set its age to 0. (**New insertion condition**) If (*a*(*t*) < *a*_*T*_) and (*h*_*b*_<*f*_*T*_) and (*c*_*b*_>*m*_*T*_) then: Add a new neuron r (*A* = *A*∪{*r*}) with a weight vector: **w**_*r*_ = 0.5·(**x**(*t*)+**w**_*b*_), *h*_*r*_ = 1. Update edges: *E* = *E*∪{(*r, b*), (*r, s*)} and *E* = *E*/{(*b, s*)}, where *a*(*t*) is the BMU's activation computed following Equation 2, *h*_*b*_ its habituation, and *c*_*b*_ its misclassification counter. If no new neuron is added: Update BMU and its neighbors *i*: (7) $$\begin{array}{r} \begin{array}{c} \Delta {\text{w}}_{b}=\epsilon_{b}\cdot h_{b}\cdot ||\text{x}\left(t\right)-{\text{w}}_{b}||,\\ \Delta {\text{w}}_{i}=\epsilon_{i}\cdot h_{i}\cdot ||\text{x}\left(t\right)-{\text{w}}_{i}||, \end{array} \end{array}$$ where the learning rates are 0 < ϵ_*i*_ < ϵ_*b*_ <1. Increment the age of all edges connected to *b* by 1. (**Introduced symbolic connections**) Update the symbolic connection weight between the BMU and the target label **y**(*t*) following Equation 3. (**New insertion condition**) If *l*_*b*_≠**y**(*t*), increase the misclassification counter: *c*_*b*_ = *c*_*b*_+1. Reduce the firing counters of the best-matching neuron and its neighbors *i*: (8) $$\begin{array}{r} \Delta h_{b}=\tau_{b}\cdot \kappa \cdot \left(1-h_{b}\right)-\tau_{b},\Delta h_{i}=\tau_{i}\cdot \kappa \cdot \left(1-h_{i}\right)-\tau_{i} \end{array}$$ with constant τ and κ controlling the curve behavior. Remove all edges with ages larger than a pre-defined threshold and remove neurons without edges. If the stop criterion is not met, repeat from step 2.

**Figure 3:**
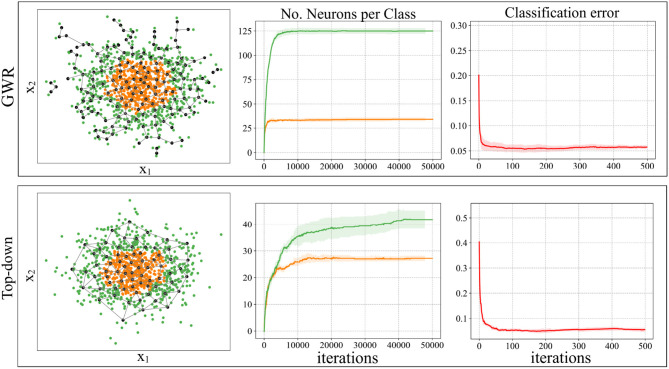
An experiment with a 2D dataset of two nested clusters demonstrates the effects of learning with a standard GWR network (first row) compared to the new neuron insertion strategy (second row) (Mici, 2018). The first plot of both rows shows the neuron placement after a complete training, the second plot shows the class-specific neural growth during each learning iteration, and the third shows the classification error measured every 100 iterations.

Figure 3 illustrates an example of the neuron placement when using the new neuron insertion strategies during classification. The dataset used for training the models is composed of one thousand data samples, drawn from a two-dimensional normal distribution, arranged in two nested clusters. The exact same parameters were used in each experiment: *f*_*T*_ = 0.3, *a*_*T*_ = 0.9, ϵ_*b*_ = 0.1, ϵ_*i*_ = 0.01, 50 training epochs and maximum edge age 50. We set a misclassification threshold *m*_*T*_ = 10. As can be easily noted in Figure 3, the neurons in a GWR algorithm try to cover the whole data distribution in the best way possible, no matter what class each data point belongs to. The use of the new neuron insertion criteria leads to the creation of a significantly smaller number of neurons, which are distributed evenly over the data samples. In the case of the original GWR, the neural growth stops way before the first 10, 000 learning iterations, i.e., the first epoch, and doesn't change much during training. The effect of applying the new neuron insertion conditions is, in this example, slightly different. We can observe a smaller number of neurons created for each class and the growth does not halt but tries to counteract the classification error during training. The overall classification error, on the other hand, remains similar to the original GWR.

## 4. Experimental Results

**Figure 4:**
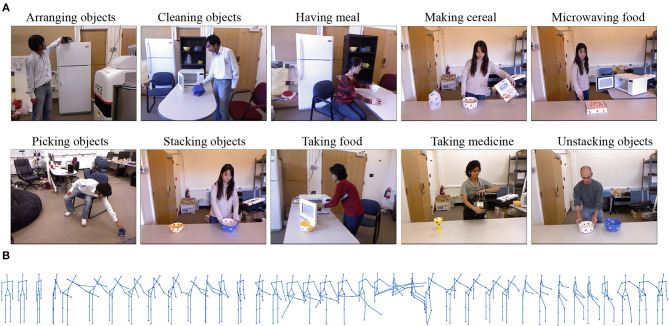
The CAD-120 dataset. **(A)** Examples of high-level activities from the dataset. **(B)** A skeleton sequence representing a person standing behind a table and microwaving food: the legs and feet have a very high tracking noise in this position due to not being visible (Mici, 2018).

We run experiments with the publically available benchmarking dataset of human activities, CAD-120. This dataset provides 120 videos of 10 long daily activities composed of a varying number of actions (Figure 4A). The dataset is challenging in the following aspects: (1) The activities in the dataset are performed by four different actors, who behave quite differently, e.g., using their left or right hand or following a different order of actions. (2) There is a large variation even for the same activity, e.g., the action *opening* can refer to opening a bottle or opening the microwave. Although both of them have the same label, they appear significantly different from each other in the video. (3) Occlusion is a critical issue for this dataset, e.g., in some of the videos, legs are occluded by the table, leading to completely unreliable leg tracks (see Figure 4B).

Since in this set of experiments we will use the object motion information provided by the dataset, an additional issue is presented by the objects being occluded by other objects (e.g., the pizza box is not tracked while inside the microwave) or not being tracked due to their small size, e.g., the apple object appearing in the *having meal* activity. This means that object location annotations provided by the dataset are often unreliable.

### 4.1. Feature Extraction

In order to process the body pose information, we extract the *skeletal quad* features (Evangelidis et al., 2014), which are invariant with respect to location, viewpoint as well as body-orientation. We consider only the position of the upper body joints (*shoulders, elbows, hands*, center of *torso, neck*, and *head*). They carry more significant information about the human-object interactions we focus on this paper than, for instance, the *feet* and the *knees* joints. We compute the positions of the hands and elbows with respect to the torso center and the neck joints by selecting two quadruples of joints: [*center torso, neck, left hand, left elbow*] and [*center torso, neck, right hand, right elbow*] and following the skeletal quad features method. We choose the neck instead of the head position due to the noisy tracking of the head caused by occlusions during actions such as *eating* and *drinking*.

From the RGB object images segmented at the beginning of each video sequence, we extract dense SIFT features with four different window sizes in order to achieve scale invariance between images. We relax the descriptors' invariance with respect to the objects' rotation by fixing the orientation of each of these descriptors. With this kind of object representation, the neurons of the trained *GWR*_*o*_ network are invariant to translation and scale, yet tuned to different object views. Then, we apply the Vector of Locally Aggregated Descriptors (VLAD) (Jegou et al., 2012) encoding method. This set of body and manipulated object visual features has been previously successfully applied to the problem of human-object interaction recognition with a self-organizing architecture (Mici et al., 2018c).

### 4.2. Adding Objects' Motion and Spatial Relationships

The recognition of human activities can be guided by the information regarding the objects involved and the way their spatial relationships change over time. For instance, putting a pizza box inside the microwave indicates that the person is microwaving food or bringing the cup toward the mouth indicates that the person is drinking. The use of objects' spatial relationships as visual features, though, raises an important question: How can such features be invariant to the scene despite the varying number and type of objects appearing in it?

**Figure 5:**
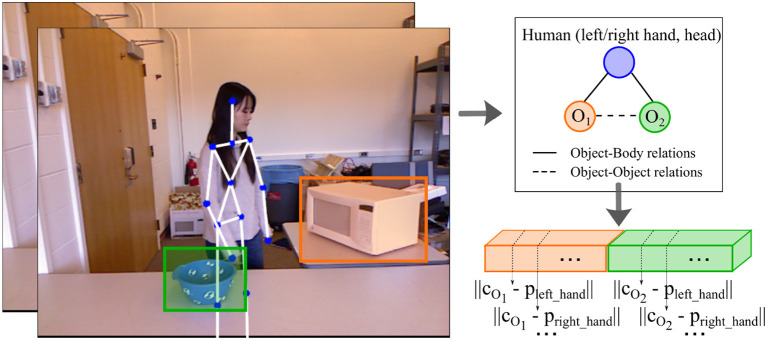
An illustration of how we represent the spatial relationships between objects and humans in a scene from the CAD-120 dataset (Mici, 2018). We extract the three-dimensional centroids of the objects, *c*_*O*_1__ and *c*_*O*_2__, and compute the Euclidean distance between them and the *left hand, right hand*, and *head* joints. This is then concatenated to the Euclidean distances between the objects' centroids. In this example, the person first interacts with the microwave and then with the bowl. Hence, the tracks of the microwave will take the first place in the concatenated vector of the spatial relationships.

One way to represent object relationships is through the scene graphs proposed by Aksoy et al. (2017). However, their approach requires the manual definition of discrete labels for spatial relations. In contrast, our goal is to keep continuous position values. Thus, we take the tracked position of the objects and form a vector whereby the order is given by the manipulation order, e.g., if the activity sequence is composed of *opening* (microwave) → *moving* (bowl) into the microwave, then the object motion vector will contain the microwave tracks concatenated to the bowl's tracks (see Figure 5). From the *x, y* coordinates given in pixels for the left upper corner and right bottom corner of the bounding boxes surrounding each tracked object, we extract the three-dimensional centroids from the corresponding depth image patches. We capture the body-object relationships by computing the Euclidean distance between the centroid of the objects to the left hand, right hand, and the head joints of the body skeleton. The object-object relationships are computed as the Euclidean difference between the three-dimensional object centroids. To capture the objects' motion information, we compute the mean velocity and the displacement of the object's centroid along the *x, y*, and *z* axis across consecutive video frames.

It should be noted that our representation of the objects' motion and spatial relationship comprises only a fraction of the input features used by the related work on the CAD-120 dataset. This is due to the fact that the input features provided by the dataset authors are suitable for learning with graphical models, such as the conditional random field (CRF) model. For instance, some features about the objects' relative positions are provided for the first, middle and the last frame of the temporal segments, which are extracted before training the model. Unlike these methods, both the learning and the recognition phase of our architecture are performed on a continuous stream of input data and no prior temporal segmentation of the actions is necessary.

### 4.3. Impact of the Top-Down Modulation During Training

Now we evaluate our architecture by running experiments with the CAD-120 dataset under two conditions: (1) considering only the architecture's feedforward connections and using the standard GWR neuron insertion strategy, and (2) considering both feedforward and top-down connections, thus applying the proposed neural growth modulation mechanism. For the first experimental setup, the architecture is trained through the hierarchical learning strategy described in section 3.3, thus the training remains *unsupervised*. For the second setup, at each learning iteration, the delayed classification errors of the activities and actions are propagated from the semantic layer to the *GWR*_*A*_ and *GWR*_*a*_ respectively, and to the network layers preceding them.

For each experimental setup, we run 4 trials, each time leaving one subject out of training, and average the obtained results. We empirically set a time window width of *q* = 30 and a lag ξ = 5 for the *GWR*_*a*_ network and *q* = 5, ξ = 1 for the *GWR*_*a*_. Thus, the first network has a temporal depth of 3 s, given that the data has a frame rate of 10 fps due to the median filter applied every 3 frames for attenuating noise. The average duration of an action in the CAD-120 dataset is around 3 s. The *GWR*_*A*_ network will have a temporal depth of 3.5 s thus developing spatiotemporal segments representing frames from at least two actions. We set a firing threshold *f*_*T*_ = 0.2, activation threshold *a*_*T*_ = 0.9, and misclassification threshold *m*_*T*_ = 4 for the *GWR*_*a*_ network and *a*_*T*_ = 0.8, and *m*_*T*_ = 2 for the *GWR*_*A*_ network.

**Figure 6:**
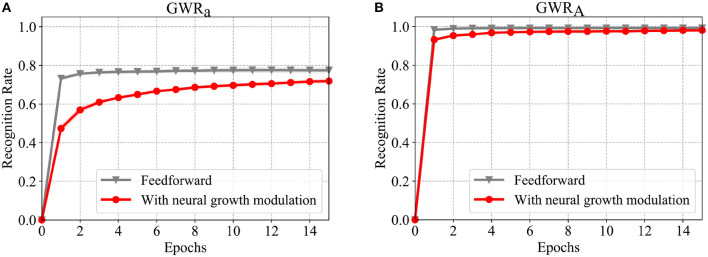
Comparison of the classification results on the training data of CAD-120 when training is conducted only with a feedforward input stream and when using the proposed neural growth modulation (Mici, 2018). **(A)** The accuracy of the *GWR*_*a*_ network which learns to classify actions, and **(B)** the accuracy of the *GWR*_*A*_ network which learns to classify the activities. The results are averaged over 4-fold cross-validation experiments.

**Figure 7:**
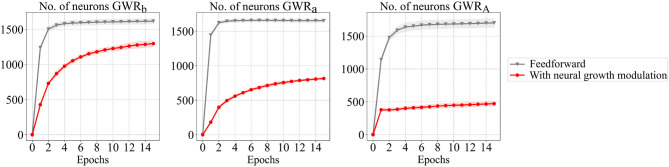
The number of neurons over the training epochs for the GWR networks with and without the top-down neural growth modulation (Mici, 2018). The results are averaged over 4-fold cross-validation experiments.

The recognition rates of the *GWR*_*a*_ and *GWR*_*A*_ networks during training for both experiments are illustrated in Figure 6. The neural growth of the body pose processing networks is illustrated in Figure 7. As can be seen from Figure 7, the number of neurons developed during learning for the second experimental setup (illustrated in red) is significantly lower than for the first setup. Most importantly, the reduced number of neurons does not compromise the classification accuracy of the activities. For the recognition of the actions, on the other hand, the experimental setup with the feedback connections results in a slightly lower accuracy. One reason for this might be the fact that the two classification errors regarding the actions and the activities are simultaneously intervening in the topographic organization of the *GWR*_*A*_ network causing this slight performance decay. Another reason might simply be that the segmentation of the actions of this dataset contains errors, thus causing higher confusion among classes. A few examples illustrating the second hypothesis will be shown in the following section.

### 4.4. Comparison With the Other Approaches

In Table 1, we report the accuracy, precision, and recall of our two models on both the actions and the high-level activities of the CAD-120 dataset. We also compare our results with the other approaches on this dataset (note that the authors of the dataset refer to the actions with the name *sub-activities*). We report both the average values of the performance measurements as well as the standard deviation across the 4 validation folds. Our model, equipped with the top-down modulation mechanism, has been listed among approaches using ground-truth segmentation due to the fact that we use the sub-activity labels during training to modulate the learning of the *GWR*_*a*_ network. The model with only feedforward connections does not use the sub-activity labels for modulating learning but associates them with each neuron for evaluation purposes. The direct comparison of the results of this table needs some caution though. The other approaches use the input features provided by the authors of the dataset, which are computed at each ground-truth temporal segment, whereas in our approach the features are computed continuously at each video frame.

**Table 1:** Classification results on the action hierarchy of the CAD-120 dataset (Mici, 2018).

**Without ground-truth segmentation**						
	**Sub-activity**			**Activity**		
**Algorithm**	**Acc. (%)**	**Prec. (%)**	**Rec. (%)**	**Acc. (%)**	**Prec. (%)**	**Rec. (%)**
Koppula and Saxena (2013), (*CRF, SVM*)	**70.3 ± 0.6**	**74.8 ± 1.6**	**66.2 ± 3.4**	83.1 ± 3.0	87.0 ± 3.6	82.7 ± 3.1
Koppula et al. (2013), (*CRF, SVM*)	68.2 ± 0.3	71.1 ± 1.9	62.2 ± 4.1	80.6 ± 1.1	81.8 ± 2.2	80.0 ± 1.2
**Hierarchical feedforward**, **(GWR)**	45.9 ± 3.8	45.0 ± 4.2	55.9 ± 7.1	**92.0 ± 3.6**	**92.5 ± 4.1**	**91.7 ± 3.7**
Rybok et al. (2014), (*SVM*)	–	–	–	78.2^*^	–	–
Tayyub et al. (2015), (*SVM*)	–	–	–	75.8 ± 6.8	77.9 ± 11.0	75.4 ± 9.1
**With ground-truth segmentation**						
Koppula and Saxena (2013), (*CRF, SVM*)	**89.3** ± 0.9	87.9 ± 1.8	**84.9 ±** 1.5	93.5 ± 3.0	95.0 ± 2.3	93.3 ± 3.1
Koppula et al. (2013), (*CRF, SVM*)	86.0 ± 0.9	84.2 ± 1.3	76.9 ± 2.6	84.7 ± 2.4	85.3 ± 2.0	84.2 ± 2.5
**Hierarchical with top-down**, **(GWR)**	43.8 ± 3.4	41.3 ± 3.1	58.6 ± 6.1	93.5 ± 3.2	94.4 ± 3.4	93.3 ± 3.3
Hu et al. (2014), (*CRF, SVM*)	87.0 ± 1.9	**89.2** ± 4.6	83.1 ± 2.4	–	–	–
Tayyub et al. (2015), (*SVM*)	–	–	–	**95.2 ± 2.0**	**95.2 ± 1.6**	**95.0 ± 1.8**

*Reported are accuracy, precision and recall (in percentage) averaged over the 4-fold cross-validation experiments*.

* *Note that Rybok et al. (2014) have not provided the standard deviation of their results. The best results are represented with bold values*.

We observed that the model with top-down connections shows a better performance regarding the classification of high-level activities and a slight decrease of the accuracy and precision on the sub-activities. Yet, the feedforward model performs better than state of the art on the high-level activities albeit the relatively low recognition accuracy on the sub-activities. This indicates that our approach does not require a fine-grained manual segmentation and a successful recognition of the actions in order to correctly classify high-level activities. The reasons for the low accuracy on the sub-activities for both models need to be further investigated.

Finally, in comparison with the other approaches in Table 1, the proposed feedforward model seems more advantageous than the model with the top-down modulation. However, for applications where human activities need to be learned incrementally during the lifetime of an intelligent agent, the second model provides a trade-off between high recognition rates and the optimization of the neural resources.

### 4.5. Learning the Activity's Compositionality

**Figure 8:**
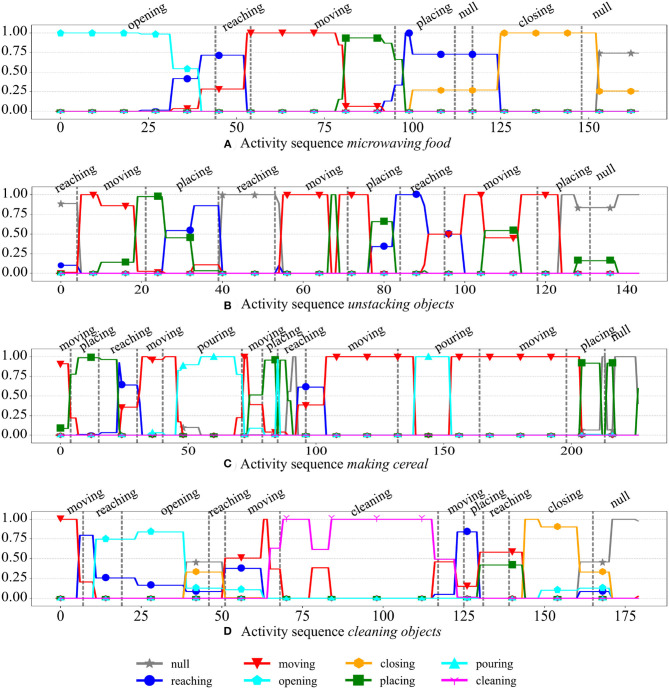
Examples of output labels of the *GWR*_*a*_ network (actions) for the test subject 1 of the CAD-120 dataset (Mici, 2018). The ground-truth temporal segmentation of the actions is illustrated with vertical gray dashed lines. The ground-truth action labels are reported on top of each plot.

We visually analyzed the output labels of the *GWR*_*a*_ network of the feedforward model during testing on *unseen* activity sequences. In Figure 8, we illustrate some examples from the subject 1. Each subfigure illustrates one activity sequence and the frame rate is of 10 fps. The ground-truth temporal segmentation provided by the dataset is depicted with vertical gray dashed lines and each plotted line interpolates the output of the best-matching neuron representing each video frame. An output of 1 indicates that the BMU has *one* Hebbian connection with a non-zero weight toward that particular category label, whereas multiple lines indicate that the BMU is connected to multiple category labels in the semantic layer. The second case happens when the neuron has matched spatiotemporal segments belonging to different categories during training and this may be due to either the similarity in the feature space of these segments, the incorrect manual segmentation of the actions or the pre-defined temporal window including several actions in it. The second reason is not to be excluded given that the segmentation of the actions in this dataset is particularly fine-grained. In Figure 8C, for instance, we can see that the activity *making cereal* is composed of 10 actions in only 100 video frames (corresponding to 10 s). There is a considerable overlap between the actions of *reaching, moving*, and *placing*. These actions compose more than half of the instances of the CAD-120 dataset.

From the examples reported in Figure 8 we can also observe the different temporal borders between the recognized actions and the ground-truth segmentation. Again, the correctness of the ground-truth segmentation plays a role here. In Figure 8A, for instance, the sequence of actions is *opening* (microwave), *reaching* (for an object), *moving* (the object), *placing* (the object inside the microwave), *null* (no action), *closing* (the microwave), and then *null*. In this example, the ground-truth segmentations do not take the action *reaching* (the microwave) into account, which is, for instance, not the case in Figure 8D where there is *reaching* and then *opening* (the microwave). In the example reported in Figure 8A, however, although with incorrect temporal boundaries, the sequence of output labels from our model is plausible.

## 5. Conclusions and Future Work

In this paper, we presented a hierarchical self-organizing architecture for the compositional learning of the human-object interactions. The architecture builds on top of our previous work (Mici et al., 2018c), and is further equipped with a top-down mechanism for the modulation of the neural growth of each body feature processing GWR network of the hierarchy. In particular, we focused on analyzing in detail the learning effects of the proposed top-down mechanism by conducting experiments with both synthetic data as well as a real-world dataset composed of human-object interactions. Overall, we observed that the application of this mechanism can lead to the creation of a considerably low number of neurons and to a higher concentration of neurons in the areas where classification is harder.

The experimental results with the CAD-120 dataset demonstrate that the proposed architecture outperforms the state-of-the-art approaches with respect to the classification of the high-level activities. The experiments also show that the average recognition accuracy for the actions is lower than in the other approaches and we analyzed a few possible reasons for this. Unlike the other methods, the proposed architecture operates on a continuous stream of information and the temporal boundaries between actions are certainly hard to determine. However, this seems to not affect the overall activity classification performance indicating that our approach is not sensitive to the correct manual segmentation and classification of the actions. Moreover, a qualitative analysis of the action labels generated by the architecture on the test data sequences showed that semantically meaningful representations had emerged. Thus, the reported results motivate further applications of the proposed architecture on other datasets for the learning of the compositionality of human activities.

The sliding time window applied in the current architecture allows us to define an arbitrary memory depth of the neurons at each level of the hierarchy, i.e., how far into the past the internal memory of each neuron stores information. In this way, we can learn short actions by setting a lower time window than when learning higher-level human activities. A similar behavior can be obtained by applying a Gamma memory instead of the sliding time window computed at the output of each GWR layer. The γ-GWR (Parisi et al., 2017b) models have an arbitrary number of temporal context descriptors and can thus be used in a hierarchical arrangement similar to the one presented in this paper. However, in both cases, the time window width hyper-parameter needs to be fine-tuned according to the learning dataset. For this reason, extensions of the GWR algorithm with variable temporal windows or temporal context descriptors should be addressed in our future work.

In this paper, we have considered only the visual stimuli of human activities. However, there are certain human-object interactions which cannot be perceived relying only on vision, e.g., a person turning on an oven or boiling water with a kettle. One approach to tackle this limitation is to add other sources of information, such as the sound generated by the object. Multimodal learning of human activities has gained a lot of interest in recent years (Stork et al., 2012; Teo et al., 2012). However, the audio-visual recognition of object manipulation actions has so far remained an open challenge (Pieropan et al., 2014b). To address this challenge, our architecture can be extended with an additional associative learning mechanism that establishes connections between networks processing auditory and visual stimuli in an unsupervised fashion (Parisi et al., 2017a).

Finally, the demonstrated capability of GWR-based hierarchical models to generate learned sequences, for instance, through lateral connections (Parisi et al., 2017a; Mici et al., 2018b), motivates the extension of our current approach toward a model that generates action strategies given a goal or a high-level activity. An interesting question would then be how to extend the proposed top-down modulation mechanism in order to retrieve the ordered sequence of actions composing an activity.

## Data Availability

The dataset used for this study is a publically available dataset. The experimental results and code for this study are available on request to the corresponding author.

## Ethics Statement

Written informed consent was obtained from the individual(s) for the publication of any potentially identifiable images or data included in this article.

## Author Contributions

LM and GP contributed to the conception and the design of the study. LM implemented the experiments and performed the statistical analysis and wrote the draft of the manuscript. SW provided approval for the publication of the content. All authors contributed to the manuscript's revision. They read and approved the submitted version.

### Conflict of Interest Statement

The authors declare that the research was conducted in the absence of any commercial or financial relationships that could be construed as a potential conflict of interest.
